# Infection and Autoimmunity

**DOI:** 10.3201/eid1203.051409

**Published:** 2006-03

**Authors:** John S. McDougal

**Affiliations:** * Centers for Disease Control and Prevention, Atlanta, Georgia, USA

**Keywords:** Book Review, Infection and Autoimmunity, Yehuda Shoenfeld, Noel R. Rose,

As the editors imply in their introduction, the relationship of infection and autoimmunity is complex, compelling, and best viewed as a physiologic process and potential consequence of normal immune recognition and immunoregulation. The editors boldly state that reading the chapters in this book brings one to the conclusion that all autoimmune diseases are infectious, until proven otherwise (my paraphrase). Add environmental triggers to the mix, and most investigators would agree.

The book is divided into 3 broad sections: mechanisms of autoimmunity; specific infectious agents and their associated autoimmune diseases; and, conversely, specific autoimmune diseases and their associated infectious agents. The chapters in the mechanisms section focus on particular mechanisms, and with 1 exception, are scholarly and well done. However, this section lacks a review or balanced discussion of the various mechanisms of autoimmunity and proof of causation. Fortunately, the first article in the pathogen section by Denman and Rager-Zisman provides an excellent overview. As with any compendium (56 chapters by more than 100 authors), the quality varies, but all are written by investigators who have made substantial contributions to the field. The book is recommended for clinical investigators with some background in infectious disease or immunology as a starting point and ready resource for the current state of knowledge in the field.

